# Patch and matrix striatonigral neurons differentially regulate locomotion

**DOI:** 10.21203/rs.3.rs-4468830/v1

**Published:** 2024-06-24

**Authors:** Huaibin Cai, Jie Dong, Lupeng Wang, Breanna Sullivan, lixin sun, Lisa Chang, Victor Martinez Smith, Jinhui Ding, Weidong Le, Charles Gerfen

**Affiliations:** National Institutes of Health; National Institutes of Health; National Institutes of Health; National Institutes of Health; National Institute on Aging, National Institutes of Health; National Institute on Aging, National Institutes of Health; National Institutes of Health; National Institute on Aging, National Institutes of Health; Dalian Medical University; NIH

**Keywords:** dorsal striatum, striosome, matrix and patch compartments, spiny projection neuron, Kremen1, substantia nigra, dopamine, aldehyde dehydrogenase 1a1 (ALDH1A1), nigrostriatal dopaminergic neurons, locomotion, GABA-B receptor (Gabbr1)

## Abstract

The striatonigral neurons are known to promote locomotion^1,2^. These neurons reside in both the patch (also known as striosome) and matrix compartments of the dorsal striatum^3–5^. However, the specific contribution of patch and matrix striatonigral neurons to locomotion remain largely unexplored. Using molecular identifier *Kringle-Containing Protein Marking the Eye and the Nose* (*Kremen1*) and *Calbidin* (*Calb1*)^6^, we showed in mouse models that patch and matrix striatonigral neurons exert opposite influence on locomotion. While a reduction in neuronal activity in matrix striatonigral neurons precedes the cessation of locomotion, fiber photometry recording during self-paced movement revealed an unexpected increase of patch striatonigral neuron activity, indicating an inhibitory function. Indeed, optogenetic activation of patch striatonigral neurons suppressed locomotion, contrasting with the locomotion-promoting effect of matrix striatonigral neurons. Consistently, patch striatonigral neuron activation markedly inhibited dopamine release, whereas matrix striatonigral neuron activation initially promoted dopamine release. Moreover, the genetic deletion of inhibitory GABA-B receptor *Gabbr1* in Aldehyde dehydrogenase 1A1-positive (ALDH1A1^+^) nigrostriatal dopaminergic neurons (DANs) completely abolished the locomotion-suppressing effect caused by activating patch striatonigral neurons. Together, our findings unravel a compartment-specific mechanism governing locomotion in the dorsal striatum, where patch striatonigral neurons suppress locomotion by inhibiting the activity of ALDH1A1^+^ nigrostriatal DANs.

Striatal spiny projection neurons (SPNs) constitute approximately 95% of the neuronal population in the dorsal striatum and play a key role in motor learning, decision-making, and regulating voluntary movement^1,2^. These SPNs can be broadly classified into two main subtypes: the dopamine receptor D1 (Drd1)-expressing direct-pathway neurons (dSPNs), which project directly to the *globus pallidus internus* (GPi) and *substantia nigra pars reticulata* (SNr); and the dopamine receptor D2 (Drd2)-expressing indirect-pathway SPNs (iSPNs), which project to the *globus pallidus externus* (GPe)^1,2^. The dSPNs primarily project to the SNr and these SNr-projecting dSPNs are also referred as striatonigral neurons. It has been well recognized that the dSPNs facilitate movement initiation and the iSPNs restrain unwanted movements^2^. Additionally, the SPNs can be further subdivided into two complementary compartments within the dorsal striatum, namely the patch and matrix compartments, identified by distinct neurochemical markers and input-output connectivity^3–5,7^. The patch compartment is typically marked by the expression of m-opiate receptor (MOR1) in rodents^3,8^, while the matrix compartment is labeled by the CALB1 expression^6^. Each compartment contains both dSPN and iSPNs. Advances in bulk and single-cell transcriptomics provide additional genetic markers for dSPNs and iSPNs as well as patch and matrix SPNs^9,10^. Previous research has also underscored the significance of patch SPNs in modulating mood, decision-making, and reward processing, while matrix SPNs are implicated in action selection^11^. However, the specific roles of patch and matrix dSPNs in locomotion control remain to be determined. Both patch and matrix dSPNs innervate the aldehyde dehydrogenase 1A1-positive (ALDH1A1^+^) dopaminergic neurons (DANs) located in the ventral tier of *substantia nigra pars compacta* (SNc)^12^. ALDH1A1^+^ DANs are particularly vulnerable to degeneration in Parkinson’s disease^13^. Notably, a portion of patch dSPN axon terminals bundle up to form distinctive striosome-dendron bouquet structures, intertwining with the dendrites of ALDH1A1^+^ DANs that extend into the SNr, in contrast to the matrix dSPNs^14,15^. This anatomical arrangement likely underlies the more potent presynaptic inhibition of DANs by patch dSPNs in contrast to matrix dSPNs^16^. Moreover, patch dSPNs exert a prolonged inhibitory influence on DAN neuronal activity via GABA-B receptors, crucially regulating the transition from tonic to burst ring in ALDH1A1^+^ nigrostriatal DANs, as evidenced by brain slice recordings^14,17^. Despite these insights, the physiological and behavioral implications of this distinctive striatonigral circuit remain to be explored.

One major challenge to study patch SPNs is the difficulty in definitively characterizing and manipulating these neurons, largely due to their less well-defined neurochemical organizations. In this study, we first identified the *Kremen1* gene as a specific molecular maker for patch SPNs in the dorsal striatum. Subsequently, we generated a line of *Kremen1*^2A-Cre^ knock-in (KI) mice to investigate the distribution of *Kremen1*-positive (*Kremen1*^+^) patch dSPN subpopulations and their functional significance in locomotor control compared to the *Calb1*-positive (*Calb1*^+^) matrix dSPNs.

## *Kremen1* transcript is enriched in patch compartments

**Figure 1:**
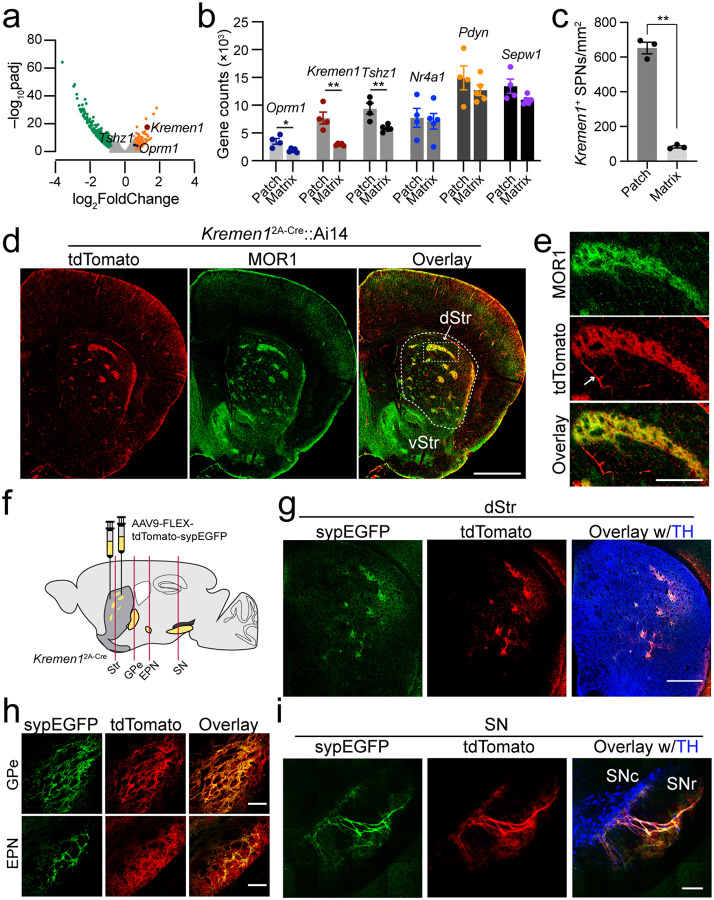
*Kremen1* labels patch neurons in the dorsal striatum (linked to Extended Data Figures 1 to 5) (**a**) Volcano plot shows differential gene expression in patch (n = 4 mice) and matrix (n = 5 mice) compartments, of which *Kremen1*, *Oprm1*, and *Tshz1* are enriched in patch neurons. (**b**) Bar graph compares the expression levels of selective genes in patch and matrix compartments. Data were presented as mean ± SEM. Unpaired t test, two tailed, *p<0.05, **p<0.01. (**c**) The densities of *Kremen1*^+^ SPNs in the patch (652.8 ± 32.94 cells / mm^2^) and matrix (83.32 ± 6.94 cells / mm^2^) compartments. N = 3 mice. Data were presented as mean ± SEM. Unpaired t test, two tailed, **p<0.01. (**d**) Representative images of tdTomato (red) MOR1 (green) in the dorsal striatum of *Kremen1*^2A-Cre^;Ai14 mice. dStr: dorsal striatum. vStr: ventral striatum. Scale bar: 1mm. (**e**) Enlarged images of the boxed region in (**d**). Arrow points to pericytes that also express tdTomato. Scale bar: 200 μm. (**f**) Schematics of AAV9-FLEX-tdTomato-synEGFP injection in the dorsal striatum of *Kremen1*^2A-Cre^ mice. The red vertical lines indicate the location of coronal sections in **g-i**. (**g-i**) Images of tdTomato (red), sypEGFP (green) and TH (blue) staining in dorsal striatum (dStr), *globus pallidus externus* (GPe), entopeduncular nucleus (EPN), and *substantia nigra* (SN). Scale bar: 500 μm in dStr, 200 μm in GPe and EPN, 100 μm in SN.

Using patch reporter *Nr4a1*-eGFP transgenic mice^18^, we isolated the enhanced green fluorescent protein (EGFP)-containing and the adjacent tissues in the dorsal striatum by laser capture microdissection for bulk RNA sequencing (**NCBI accession: PRJNA870469**). In comparison to various patch markers examined previously, such as *mu-type Opioid Receptor* (*Oprm1*), *Teashirt Zinc Finger Family Member 1* (*Tshz1*), *Prodynorphin* (*Pdyn*)and *Selenoprotein W* (*Sepw1*), the expression of *Kremen1* mRNA exhibited substantially higher differentials in the patch versus matrix compartments (Fig. 1a, b). Consistently, *Kremen1* was also identified as a patch marker by an independent single-nuclei transcriptomic study^10^. Further co-localization analyses with *Drd1* and *Drd2* using RNAscope *in situ* hybridization found that approximately 60% of *Kremen1*^+^ SPNs corresponded to dSPNs, while 40% were identified as iSPNs, resembling the ratio of total dSPNs versus iSPNs in the dorsal striatum (**Extended data Fig. 1a-c**). Moreover, *Kremen1*^+^ SPNs were predominantly distributed in the dorsal striatum (Fig. 1d, **Extended data Fig. 1d**). Within the striatum, *Kremen1*^+^ SPNs accounted for 12.7% of total SPNs, 52% of SPNs within the patch compartments, and 8% of SPNs in the matrix compartments (**Extended data Fig. 1e**). Notably, the density of *Kremen1*^+^ SPNs was substantially higher in patch compartments compared to matrix compartments (Fig. 1c). Together, these findings identify *Kremen1* as a promising genetic marker for distinguishing patch-matrix compartments.

**Figure 2:**
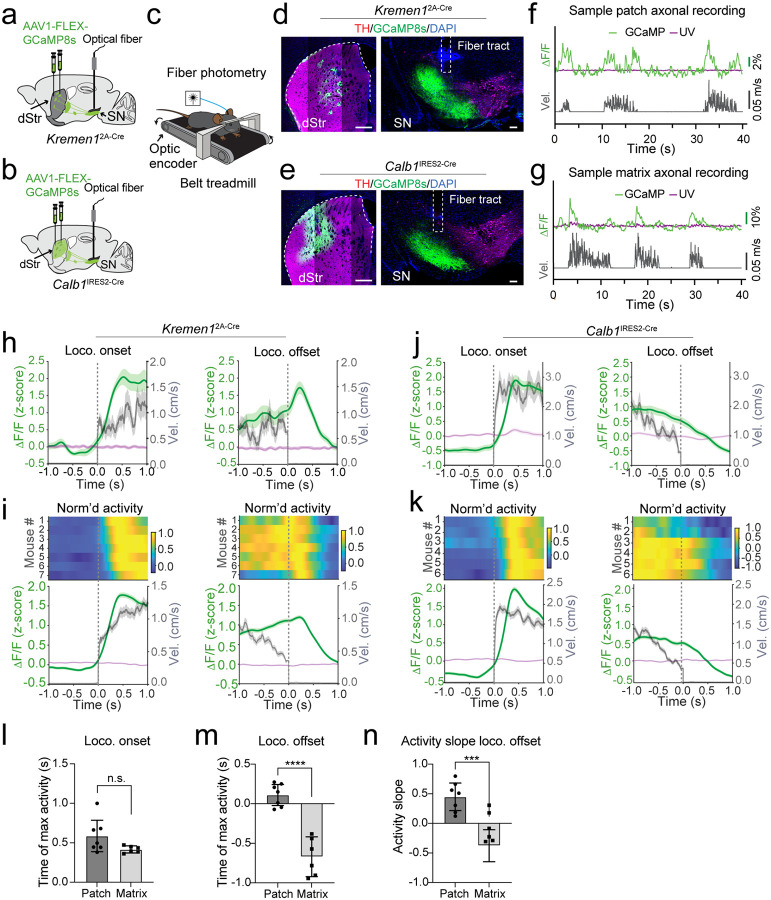
Activity of patch and matrix dSPNs during self-paced locomotion (**a, b**) Schematics outlines AAV injection and optic fiber implantation for recording the activity of patch (**a**) and matrix dSPNs (**b**). (**c**) Cartoon shows a head-fixed mouse with optic fiber implantation on a belt treadmill. (**d, e**) Images of GCaMP8s (green), TH (magenta), and DAPI (blue) in the dStr and SN of *Kremen1*^2A-Cre^ (**d**) and *Calb1*^IRES2-Cre^ mice (**e**). Fiber track is indicated in the SN. Scale bar: 500 μm (dSTR) and 100 μm (SN). **(f, g**) Sample traces of GCaMP8s (green) and UV isosbestic (purple) with corresponding velocity (gray) traces from a representative *Kremen1*^2A-Cre^ (**f**) and a *Calb1*^IRES2-Cre^ mouse (**g**). (**h, j**) Alignments of the activity patch (**h**) and matrix dSPNs (**j**) activity during locomotion onset (left) and offset (right) from the sample recordings in **f** and **g**. (**i, k**) The top heat-maps show normalized mean patch (**i**) and matrix (**k**) dSPN activity of individual mice. The bottom graphs depict population mean patch (**i**, n = 7 mice) and matrix (**k**, n = 7 mice) dSPN activity across animals aligned on locomotion onset (left) and offset (right). (**l**) Timing of maximum activity during locomotion onset. Unpaired t test, two tailed, p = 0.08. (**m**) Timing of maximum activity during locomotion offset. Unpaired t test, two tailed, ****p < 0.0001. (**n**) Slope of activity preceding locomotion offset. Unpaired t test, two tailed, ***p< 0.001. All error bars in this figure were represented as mean ± SEM.

*Tshz1* is also enriched in patch compartments^9,10^. We further compared the distribution of *Kremen1*^+^ and *Tshz1*^+^ dSPNs and total SPNs in the dorsal striatum by RNAScope *in situ* hybridization and found only a 7–8% overlap between these two dSPN subpopulations (**Extended Date Fig. 2**). These results suggest that *Kremen1*^+^ and *Tshz1*^+^ mark two largely different subtypes of patch SPNs in the dorsal striatum.

## *Kremen1*^2A-Cre^ knock-in mice are useful for studying patch SPNs

Given the enrichment of *Kremen1* in patch SPNs, we generated a line of *Kremen1*^2A-Cre^ knock-in (KI) mice using CRISPR/Cas9-mediated gene editing (**Extended data Fig. 3**). By crossing these mice with Ai14 reporter mice, which express the red fluorescent protein tdTomato under Cre-dependent regulation, we observed distinct tdTomato signals scattered within the dorsal but not ventral striatum of *Kremen1*^2A-Cre^;Ai14 mice (Fig. 1d). These signals co-localized with the widely used patch marker MOR1 (Fig. 1d, e). Apart from patch SPNs, tdTomato signals were also detected in pericytes, with no staining detected in the interneurons or glial cells of the dorsal striatum (**Extended data Fig. 4a, b**). The *Kremen1*^+^ patchy structures were restricted to the dorsal striatum but distributed throughout the entire dorsal striatum along the rostral to caudal and medial to lateral axes (**Extended data Fig. 5a, b**). Beyond the dorsal striatum, tdTomato signals were also visible in the hippocampal regions (**Extended data Fig. 5b, c**).

To investigate the projection pattern of *Kremen1*^+^ SPNs, we employed stereotaxic injection of an adeno-associated viral vector (AAV) co-expressing Cre-dependent tdTomato and synaptophysin-fused EGFP (sypEGFP) into the dorsal striatum of *Kremen1*^2A-Cre^ KI mice (Fig. 1f). Both tdTomato and sypEGFP signals were found in the patch compartments (Fig. 1g). Furthermore, sypEGFP specifically marked the axon terminals of patch SPNs in the GPe and entopeduncular nucleus (EPN), a mouse equivalent to GPi (Fig. 1h), as well as in the SNr and SNc (Fig. 1i). Notably, the incoming tdTomato-positive patch dSPN axons exhibited distinct dendron-bouquet like structures^15^, forming connections with the dendrites of DANs in SNr (Fig. 1i). This striosome-dendron bouquet structure was also observed in the SN of *Kremen1*^2A-Cre^;Ai14 mice (**Extended data Fig. 5d**). In summary, *Kremen1*^2A-Cre^ KI mice may serve as a useful tool for investigating the neuroanatomy and functions of patch SPNs. Therefore, *Kremen1*^+^ SPNs will be referred to as patch SPNs hereafter.

## Increased patch dSPN activity precedes the cessation of locomotion

To monitor the activity of patch and matrix dSPNs during self-paced locomotion, we injected AAVs expressing the genetically encoded calcium indicator GCaMP8s (AAV1-FLEX-GCaMP8s) into the dorsal striatum and implanted an optic fiber in the SNr of the same hemisphere in *Kremen1*^2A-Cre^ or *Calb1*^IRES2-Cre^ mice^19^ (Fig. 2a, b). The mice were head-fixed and allowed to walk freely on a belt treadmill, where movement velocity signal was synchronized with a fiber photometry setup for recording calcium transients in the axon terminals of patch or matrix dSPNs simultaneously (Fig. 2c–e). As shown in representative sample recording traces from a *Kremen1*^2A-Cre^ and a *Calb1*^IRES2-Cre^ mouse respectively, both patch and matrix dSPN axon terminal calcium transients covaried with velocity changes during locomotion bouts (Fig. 2f, g). However, when aligning their mean activity with locomotion onset and offset, it revealed a difference between patch and matrix dSPNs. In the same sample mice, both patch and matrix dSPN activity increased following the onset of locomotion, but there was an unexpected rise of patch dSPN activity preceding the locomotion cessation and continued to rise until shortly after animal stopped moving, in contrast, the activity of matrix dSPN decreased preceding the locomotion cessation (Fig. 2h, j). This pattern was consistent with additional analyses involving more animals (Fig. 2i, k). The timing of peak activity during locomotion onset did not significantly differ between patch and matrix dSPNs (p = 0.08, two-tailed unpaired t test, Fig. 2l). However, the timing of peak activity during locomotion offset was notably delayed in patch dSPNs compared to matrix dSPNs (p < 0.0001, Fig. 2m), and in fact patch dSPN activity peaked after locomotion offset (p = 0.04, one-tailed one-sample t test). Additionally, the slope of activity preceding locomotion offset differed significantly between patch and matrix dSPNs (p < 0.001, Fig. 2n), with patch dSPNs exhibiting increased (positive) and matrix dSPNs showing decreased (negative) activity, suggesting their differential regulation of motor activity.

## Patch and matrix dSPNs exert contrasting roles in locomotor control

**Figure 3:**
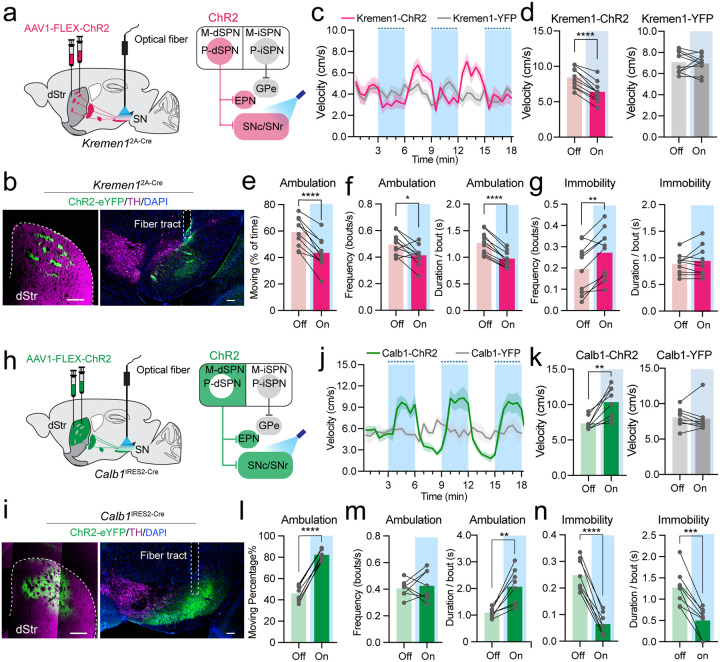
Optogenetics activation of patch and matrix dSPNs during locomotion (linked to extended Data Figures 6 and 7) (**a**) Schematics of AAV injection and optic fiber implantation for selectively activating patch dSPNs in *Kremen1*^2A-Cre^ mice. (**b**) Representative images of ChR2 (green), TH (magenta) and DAPI (blue) in the dStr and SN. The fiber implant location is marked in the SN. Scale bar: 500 μm (dSTR) and 100 μm (SN). (**c**) Instantaneous locomotion velocity in open-field test with light-off and light-on (blue shaded area) in *Kremen1*^2A-Cre^ mice. N=10 mice for each group. YFP stands for the yellow fluorescent protein control. The data were represented as mean (dark line) ± SEM (shaded area). (**d**) Average velocity during ambulation bouts in *Kremen1*^*2A-Cre*^ mice with ChR2 (Off: 8.41 ± 0.45 cm/s vs. On: 6.38 ± 0.46 cm/s) and YFP (Off: 7.10 ± 0.32 cm/s vs. On: 6.96 ± 0.28 cm/s) injection. N=10 mice for each group. Paired t test, two tailed, ****p<0.0001. (**e**) The percentage of time for ambulation (Off: 59.31 ± 3.50 % vs. On: 42.98 ± 3.68 %). Paired t test, two tailed, ****p < 0.0001. (**f**) Frequency (Off: 0.50 ± 0.03 bout/s vs. On: 0.41 ± 0.02 bout/s) and duration (Off: 1.26 ± 0.07s vs. On: 0.96 ± 0.05s) of ambulation bouts. Paired t test, two tailed, *p<0.05, ****p < 0.0001). (**g**) Frequency (Off: 0.20 ± 0.04 bout/s vs On: 0.27 ± 0.04 bout/s) and duration (Off: 0.89 ± 0.06s vs On: 0.93 ± 0.09s) of immobility bouts. Paired t test, two tailed, **p<0.01. (**h**) Schematics of AAV injection and optic fiber implantation for selectively activating matrix dSPNs in *Calb1*^IRES2-Cre^ mice. (**i**) Representative images of ChR2 (green), TH (magenta) and DAPI (blue) in the dStr and SN. The fiber implant location is indicated in the SN. Scale bar: 500 μm (dStr) and 100 μm (SN). (**j**) Instantaneous locomotion velocity in open-field test with light-off and light-on in *Calb1*^IRES2-Cre^ mice. N=8 for each ChR2 and YFP group. (**k**) Average velocity during ambulation bouts in *Calb1*^IRES2-Cre^ mice with ChR2 (Off: 7.37 ± 0.33 cm/s vs. On: 10.33 ± 0.79 cm/s) and YFP (Off: 8.08 ± 0.48 cm/s vs. On: 7.88 ± 0.70 cm/s) injection. N=8 mice for each group. Paired t test, two tailed, **p = 0.0042. (**l**) The percentage time for ambulation (Off: 46.01 ± 2.55 % vs. On: 82.30 ± 1.88 %). N=8 mice each group. Paired t test, two tailed, ****p < 0.0001. (**m**) Frequency of ambulation bouts (Off: 0.41 ± 0.02 bout/s vs On: 0.43 ± 0.04 bout/s) and duration (Off: 1.09 ± 0.06s vs On: 2.06 ± 0.23s). N=8 mice each group. Paired t test, two tailed, **p<0.01). (**n**) Frequency of immobility bout (Off: 0.25 ± 0.02 bout/s vs On: 0.06 ± 0.01 bout/s) and duration (Off: 1.27 ± 0.15s vs On: 0.50 ± 0.11s). N=8 mice each group. Paired t test, two tailed, ***p<0.001, ****p<0.0001. All error bars in this figure were represented as mean ± SEM.

To examine the influence of patch dSPN activity on locomotion, we used optogenetic manipulations by introducing AAV vectors containing Cre-dependent optogenetic activator Channelrhodopsin-2 (AAV1-FLEX-ChR2) into the dorsal striatum of *Kremen1*^2A-Cre^ mice and implanted the optic fiber in the SNr for light stimulation of dSPN axon terminals (Fig. 3a, b). Optogenetic activation at 3mW/20Hz for 3min led to a notable reduction in locomotion velocity, followed by a subsequent recovery in motor activity after the stimulation period (Fig. 3c). The decrease in walking speed likely resulted from both a reduction in velocity during each bout of ambulatory movement (Fig. 3d) and reduced locomotor activity, as evidenced by both reduced movement frequency and shortened duration (Fig. 3e, f), as well as an increase in immobility frequency (Fig. 3g). This reduction in locomotion velocity was also observed at various stimulation parameters, including continuous 0.25mW power intensities, 10Hz stimulation frequency, and 10s durations (**Extended data Fig. 6a-c**). In contrast, optogenetic activation of matrix dSPNs of *Calb1*^IRES2-Cre^ mice yielded opposite outcomes, resulting in a substantial increase in movement velocity, coupled with an increase of ambulatory speed and duration, as well as a decrease in immobility frequency and duration (Fig. 3h–n, **Extended data Fig. 6d-f**). Consequently, our findings unravel the opposing roles of patch and matrix dSPNs in the regulation of ambulatory movement.

To investigate the role of patch iSPNs in the context of locomotion, we introduced AAV vectors containing optogenetic activators that were both Cre and Flp-dependent (AAV1-ConFon-ChR2) into the dorsal striatum of *Kremen1*^2A-Cre^;*A2a*^Flp^ double KI mice (**Extended data Fig. 7a**). Light stimulation was applied at the axon terminals of iSPNs situated in the GPe (**Extended data Fig. 7b, c**). The high-power stimulation at 3mW/20Hz for 3min resulted in a modest increase in average velocity and duration per bout of movement (**Extended data Fig. 7d-h**), while at low light power level with 0.25mW constant stimulation we did not observe any notable alteration in locomotion velocity (**Extended data Fig. 7i**). These results underscore the limited role of patch iSPNs in regulating locomotion. Indeed, chemogenetic activation of both patch dSPNs and iSPNs in *Kremen1*^2A-Cre^ mice led to reduced locomotor activity (**Extended data Fig. 8**), supporting a major role of patch dSPNs in suppressing locomotion.

## Patch and matrix dSPNs differently regulate dopamine release

**Figure 4:**
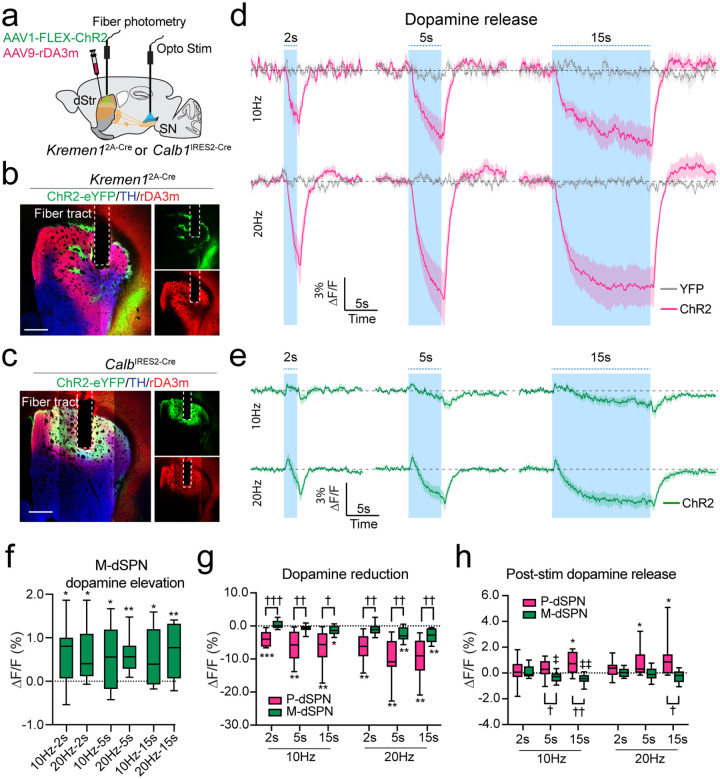
Patch and matrix dSPNs differentially regulate dopamine release (**a**) Schematics of simultaneous fiber photometry recording and optogenetics stimulation for patch and matrix dSPN-induced changes of dopamine levels in the dorsal striatum. (**b, c**) Representative images of ChR2 (green), rDA3m (red), and TH (blue) in the dStr of *Kremen1*^2A-Cre^ (**b**) and *Calb1*^IRES2-Cre^ (**c**) mice. The locations of photometric optic fibers are masked in the dStr. Scale bar: 500 μm. (**d, e**) Changes of dopamine levels in the dStr during various light stimulation frequency and duration in the SN of *Kremen1*^2A-Cre^ (**d**) and *Calb1*^IRES2-Cre^ (**e**) mice. The data represented mean of each group of mice (dark line) ± SEM (shaded area). Patch dSPN (P-dSPN)-ChR2 = 8 mice, P-dSPN-YFP = 3 mice, matrix dSPN (M-dSPN)-ChR2= 8 mice. (**f**) The amplitude of dopamine elevation 0.35 – 0.45s after stimulating M-dSPNs. N=8 mice, one sample t test, right tailed, *p<0.05, **p<0.01. (**g**) The amplitude of dopamine reduction following activation of patch (red) and matrix (green) dSPNs. N=8 mice per group, one sample t test, left tailed, * p < 0.05, ** p < 0.01, ***p < 0.001. Unpaired t test of dopamine reduction amplitude between P-dSPN-ChR2 and M-dSPN-ChR2 groups, two tailed, † p < 0.05, †† p < 0.01, ††† p < 0.001. (**h**) Post stimulation (post-stim) dopamine release after activating P-dSPNs and M-dSPNs. N=8 mice per group, for P-dSPNs, one sample t test, right tailed, * p < 0.05; for M-dSPNs, one sample t test, left tailed, ^‡^ p < 0.05, ^‡‡^ p < 0.01. Unpaired t test of post-stim dopamine release between activation of P-dSPNs and M-dSPNs, n=8 mice per group, two tailed, † p < 0.05, †† p < 0.01. Data were represented as median ± Min to Max.

To explore how patch and matrix dSPNs distinctively regulate locomotion at the circuit level, we examined the impact of dSPN neuronal activity on dopamine release in the dorsal striatum. Both patch and matrix dSPNs innervate nigrostriatal DANs and regulate their activity^12,14,16,20^. Using the genetically encoded dopamine sensor GRAB_DA3m_^21^ and Cre-dependent ChR2, we assessed dopamine release in the dorsal striatum of *Kremen1*^2A-Cre^ (Fig. 4a, b) and *Calb1*^IRES2-Cre^ (Fig. 4a, c) mice through fiber photometry following optogenetic stimulation at the SNr for each mouse line. Activation of patch dSPNs led to a gradual reduction in dopamine levels in a stimulation dose dependent manner, with stimulation frequencies of 10 Hz and 20 Hz, and durations of 2, 5, and 15 seconds (Fig. 4d). In contrast, activation of matrix dSPNs induced a biphasic change in dopamine release: an initial transient increase in dopamine signals at the onset of optic stimulation, followed by a subsequent reduction during the stimulation (Fig. 4e). The initial surge of dopamine release following matrix dSPN activation was present across different stimulation frequencies and durations (Fig. 4f). However, the magnitude of dopamine release reduction was far less pronounced with matrix dSPNs stimulation compared to patch dSPNs (Fig. 4g). Additionally, only patch dSPN stimulation resulted in a post-stimulation rebound of dopamine release (Fig. 4h), consistent with a previous observation of DANs rebound ring following patch stimulation in brain slice^14^. These findings suggest that patch and matrix dSPNs regulate dopamine release differently, with patch dSPN activation leading to a more potent inhibition of dopamine release.

## Patch dSPNs suppress locomotion through the GABA-recs in ALDH1A1^+^ DANs

We next set to determine whether patch dSPNs affected locomotion through inhibiting nigrostriatal DANs. Considering that ALDH1A1^+^ nigrostriatal DANs receive prolonged inhibitory inputs mediated by GABA-B receptors from patch dSPNs^14,17^, we genetically deleted the receptor-encoding *Gabbr1* gene in ALDH1A1^+^ nigrostriatal DANs through introduction of Cre-dependent CRISPR/saCas9-*Gabbr1*sgRNA gene targeting AAVs into the SNc of *Aldh1a1*^CreERT2^ mice^12^ (**Extended Data Fig. 9a, b**). The resulting knockdown (KD) of Gabbr1 in ALDH1A1^+^ nigrostriatal DANs led to increased ambulatory velocity (**Extended Data Fig. 9c-e**). These findings support an important role of GABA-B receptor-mediated inhibitory signaling in ALDH1A1^+^ DANs in locomotor control.

**Figure 5:**
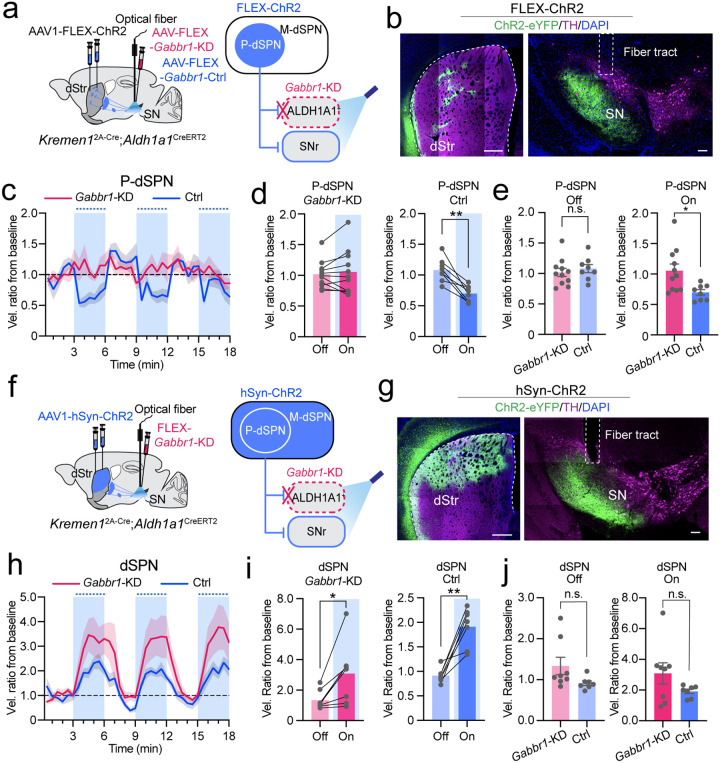
Knockdown GABA-B receptor in ALDH1A1^+^ DANs abolishes patch dSPN-induced locomotion suppression (**a**) Selective activation of P-dSPNs and genetically deletion of GABA-B receptors (*Gabbr1*-KD) in ALDH1A1^+^ DANs in *Kremen1*^2A-Cre^;*Aldh1a1*^CreERT2^ double KI mice. (**b**) Images of ChR2 (green), TH (magenta) and DAPI (blue) in the dStr and SN. Optic fiber implant location was marked in the SN. Scale bar: 500 μm (dSTR) and 100 μm (SN). (**c**) Instantaneous locomotion velocity change (from the first 3-min base line) in open-field test when activating P-dSPNs in *Gabbr1* KD and control (Ctrl) conditions. *Gabbr1* KD = 11 mice, Ctrl = 8 mice. (**d**) Comparison of velocity changes between light-off and -on periods in *Gabbr1* KD group (Off: 1.02 ± 0.07 of baseline vs. Off: 1.06 ± 0.11 of baseline) and Ctrl group (Off: 1.08 ± 0.07 of baseline vs. Off: 0.70 ± 0.05 of baseline). Paired t test, two tailed, p = 0.49 (KD), and **p = 0.001 (Ctrl). (**e**) Comparison of velocity difference between *Gabbr1*-KD and Ctrl mice during light-off and light-on periods. Unpaired t test, two tailed, p = 0.518 (light-off), *p = 0.0165(light-on). (**f**) Activation of both patch and matrix dSPNs and selective deletion of *Gabbr1* in ALDH1A1^+^ DANs in *Kremen1*^2A-Cre^;*Aldh1a1*^CreERT2^ double KI mice. (**g**) Images of ChR2 (green), TH (magenta), and DAPI (blue) in the dStr. The fiber implant location was marked in the SN. Scale bar: 500 μm (dStr) and 100 μm (SN). (**h**) Instantaneous locomotion velocity change (from the first 3-min base line) in open-field test under *Gabbr1*-KD and Ctrl condition during activation of dSPNs. *Gabbr1*-KD = 8 mice, Ctrl = 7 mice. (**i**) Comparison of velocity changes between light-off and -on periods in *Gabbr1*-KD group (Off: 1.34 ± 0.22 of baseline vs. Off: 3.08 ± 0.69 of baseline) and Ctrl group (Off: 0.92 ± 0.06 of baseline vs. 1.91 ± 0.15 of baseline). Paired t test, two tailed, *p = 0.013 (*Gabbr1*-KD), **p = 0.0013 (Ctrl). (**j**) Comparison of velocity difference between *Gabbr1*-KD and Ctrl conditions during light-off and -on periods. Unpaired t test, two tailed, p = 0.09 (light-off), p = 0.1451 (light-on). All error bars in this figure were represented as mean ± SEM.

To investigate the role of postsynaptic GABA-B receptors in patch dSPN-induced locomotor inhibition, we selectively activated the patch dSPNs through optogenetics while simultaneously downregulating *Gabbr1* in ALDH1A1^+^ nigrostriatal DANs of *Kremen1*^2A-Cre^;*Aldh1a1*^CreERT2^ double KI mice (Fig. 5a, b). Since the *Gabbr1*-KD and control mice tended to have different baseline locomotion, to compare between groups, we computed a velocity ratio from baseline for individual mice. Remarkably, the genetic KD of *Gabbr1* in the ALDH1A1^+^ nigrostriatal DANs completely abolished the locomotion suppressing effects induced by patch dSPN activation (Fig. 5c, d). Correspondingly, control mice showed more reduction in velocity compared to *Gabbr1*-KD mice during light stimulation (Fig. 5e). To further validate the involvement of GABA-B receptors in locomotion inhibition, we stimulated both patch and matrix dSPNs in *Kremen1*^2A-Cre^;*Aldh1a1*^CreERT2^ double KI mice under the control and *Gabbr1*-KD conditions (Fig. 5f, g). While the dSPN stimulation markedly increased locomotion in both groups of mice, the absence of GABA-B receptors in ALDH1A1^+^ nigrostriatal DANs seemed to further augment dSPN-induced ambulatory movement (Fig. 5h, i), although this effect did not reach statistical significance (Fig. 5j). Together, our findings demonstrate that patch dSPNs negatively modulate ambulatory locomotor behavior by regulating ALDH1A1^+^ nigrostriatal DAN activity through GABA-B receptors.

## Discussion

In this study we challenge the canonical model suggesting that striatonigral dSPNs promote locomotion and demonstrate that this model perhaps only applies to the matrix striatonigral dSPNs, while the patch striatonigral dSPNs play an opposing role by suppressing locomotion. This inhibitory function of patch dSPNs is implicated in the rise of neuronal activity during the offset of self-paced movement and is causally validated by optogenetic activation. Furthermore, we showed the critical involvement of GABA-B receptors in ALDH1A1^+^ nigrostriatal DANs during patch dSPN-induced locomotion inhibition.

The dSPNs and iSPNs are subdivided into patch and matrix compartments based on neurochemical markers and their input-output connectivity;^7^ however, it remains challenging to ambiguously define the patch compartments. Conventional patch markers, including MOR1, substance P and enkephalin, can only identify a portion of patch compartments along the rostral to caudal axis of the striatum^22^. Specifically, MOR1 marks the patch in the rostral and intermediate striatum, while substance P and enkephalin label the patch in the intermediate and caudal striatum^22^. In contrast, *Kremen1* marks the patch structures within the entire dorsal striatum, including the dorsolateral striatum, a region heavily involved in motor control and learning^23^. Additionally, *Kremen1* marks both Drd1-and Drd2-expressing SPNs in the same ratio as the general SPN population, while MOR1 tends to label more Drd1-expressing dSPNs^24^. In addition to MOR1, *Pdyn* and *Tshz1* are also preferentially mark the patch dSPNs^9,10^. Notably, there is less than 3% overlap between the *Pdyn*^+^ and *Tshz1*^+^ Drd1-expressing dSPNs^25^, suggesting heterogeneity among patch SPNs based on different gene expression pro les. Furthermore, substantially more *Pdyn*^+^ and *Tshz1*^+^ SPNs are distributed in the matrix compartments compared to the *Kremen1*^+^ SPNs. Therefore, *Kremen1* serves as a useful patch marker, which unbiasedly labels both dSPNs and iSPNs selectively in the patch compartments.

The *Pdyn*-positive (*Pdyn*^+^) dSPNs were suggested to promote locomotion, while the *Tshz1*-positive (*Tshz1*^+^) ones were claimed to suppress locomotion in a place-preference test^25^. Since patch dSPNs have been implicated in discriminating valence during decision-making, changes in movement velocity by patch dSPN activation in valence context (such as place preference test) may reflect alterations in active avoidance or approach choices rather than locomotion itself^11^. Moreover, the roles of SNr-projecting dSPNs were not specifically examined in the aforementioned study^25^, considering GPi-projecting dSPNs may originate from a distinct population^26^. As *Tshz1*^+^ patch dSPNs have also been associated with locomotion suppression^25^, we conducted a comparison of *Kremen1*^+^ and *Tshz1*^+^ Drd1-expressing neurons in the dorsal striatum. Our findings reveal that *Kremen1* and *Tshz1* mark two largely different subtypes of patch dSPNs in the dorsal striatum. While we demonstrate that SNr-projecting *Kremen1*^+^ dSPNs inhibit ambulatory locomotion, whether SNr-projecting *Tshz1*^+^ SPNs serve a similar function remains unclear^25^. Given the significant diversity among patch SPNs based on molecular markers and connectivity, future studies should explicitly identify subtypes using genetic markers and projection targets.

Both patch and matrix dSPN activity showed a similar transient increase at the onset of locomotion, but started to diverge as movement ceased, with matrix dSPN exhibiting decreased activity while patch dSPN transiently increased activity. The activity pattern of patch and matrix dSPNs during locomotion mirrors previous findings of synergistic concurrent activation of opposing dSPNs and iSPNs during movement initiation^24^ with their activity decorrelating as movement progresses^27^. Furthermore, manipulating these two populations of dSPNs causally produced opposing locomotor effects. These findings highlight the presence of opposing motifs within the dSPN population, one originating from the patch and the other from matrix, akin to the dichotomous organization of dSPN and iSPN in the broader dorsal striatum. Our results suggest that patch dSPN activity may facilitate state transition from movement to quiescent. In support of this hypothesis, patch dSPN activity began to ramp hundreds of milliseconds before movement termination, and optogenetic activation of patch dSPNs shortened movement bout duration while increasing instance of immobility. State transition regulation has been identified as a fundamental principle of basal ganglia motor control^28^. In addition to receive sensorimotor inputs, patch dSPNs receive more limbic inputs than matrix dSPNs^29^ and have robust direct connections with midbrain DANs, suggesting their role in integrating external sensorimotor information with implicit motivation to regulate motor state transitions. Conversely, we found that patch iSPNs exerted a positive, albeit less pronounced, effect on ambulatory movement upon activation compared to patch dSPNs. This aligns with previous findings suggesting that striatal iSPNs apply inhibitory motor control through their GABAergic axon collaterals to surrounding dSPNs, while the majority of striatopallidal inputs drive negative reinforcement learning^30^. The synaptic strength of iSPN-dSPN connections within the patch remains unclear. Given the relatively smaller number of patch iSPNs, their collaterals within the patch may not be as robust. Further exploration is warranted to elucidate the function of patch iSPNs.

ALDH1A1^+^ nigrostriatal DANs receive the most monosynaptic inputs from the dorsal striatum^12^. Furthermore, these neurons appear to form reciprocal innervation with dSPNs in the dorsal regions of dorsal striatum^12^. This reciprocal connection between ALDH1A1^+^ DANs and dSPNs may constitute a feedback loop for timely regulation of the dopamine release and SPN activity in motor control and learning. While both patch and matrix dSPNs innervate ALDH1A1^+^ DANs^12^, the axon terminals of patch dSPNs are the primary presynaptic components in this so-called striosome-dendron bouquet structure with the dendrites of ALDH1A1^+^ nigrostriatal DANs^14,15^. This arrangement potentially plays a pivotal role in regulating dopamine release in both dorsal striatum and SNr. Accordingly, brain slice recordings indicate that patch dSPNs deliver stronger inhibitory inputs to the DANs and could induce rebound ring in ALDH1A1^+^ nigrostriatal DANs compared to matrix dSPNs and the other inhibitory inputs^14,16^. Consistent with these *in vitro* studies, our *in vivo* recordings reveal a more pronounced inhibition of dopamine release upon stimulating patch dSPNs compared to the matrix dSPNs, with a rebound dopamine release observed upon the cessation of patch dSPN stimulation. Supporting the functional significance of the connection between patch dSPNs and ALDH1A1^+^ nigrostriatal DANs, genetic deletion of GABA-B receptors in ALDH1A1^+^ nigrostriatal DANs completely abolished the locomotion-suppressing effects mediated by patch dSPNs. This synaptic coupling might dynamically regulate the dopamine supply in both the dorsal striatum and SNr, thereby modulating various motor activities.

Notably, the activation of matrix dSPNs initially triggered a transient increase in dopamine release before a subsequent reduction occurred. Considering that matrix dSPNs project to inhibitory parvalbumin neurons (PVNs) in the SNr and PVNs provide direct and tonic inhibitory inputs to DANs^31–34^, it’s plausible that matrix dSPNs initially disinhibit DANs via PVNs, resulting in an early increase in DAN activity and dopamine release. The prevalence of this transient increase of dopamine release within the first two seconds of matrix dSPN stimulation suggests that the primary effect of matrix dSPN activation on DANs is disinhibition or excitation. Therefore, the distinct modulation of DAN activity by patch and matrix dSPNs may underlie their differentia influence on motor control.

## Methods

### Mouse work

All mouse studies were conducted in accordance with the guidelines approved by the Institutional Animal Care and Use Committees (IACUC) of the Intramural Research Program of the National Institute on Aging (NIA), NIH. All mouse lines were maintained as heterozygotes in a C57BL/6J background. The *Kremen1*^2A-Cre^ KI mice were generated by Shanghai Model Organisms Inc. (Shanghai, China). The *Aldh1a1*^CreERT2^ KI mice were generated as previously described^12^. The *Nr4a1*–eGFP (Stock No: 036737-UCD) transgenic mice were obtained from Mutant Mouse Resource & Research Centers (MMRRC). The *Calb1*^IRES2-Cre^ mice (Stock No: 028523) and Ai14 (Stock No: 007908) were obtained from the Jackson laboratory. The *A2a*^Flp^ KI mice were generated by the Rodent Transgenic Core of National Institute of Mental Health (NIMH). Both females and males were used for all experiments. Mice used for viral injections were between 2 and 4 months of age. The mice were housed in a 12-hour-light/12-hour-dark cycle in groups of 2–5 animals and had ad libitum water and a regular diet. All the behavioral tasks were performed during the light cycle. Littermates were randomly assigned to different groups prior to experiments.

### Generation of Kremen1^2A-Cre^ KI mice

The *Kremen1*^2A-Cre^ KI mice were generated using the CRIPSR/Cas9 approach in the C57BL/6J strain by Shanghai Model Organisms Inc. A donor plasmid containing the 2A ribosome skipping sequence, Cre DNA recombinase sequence, and flanking *Kremen1* mouse genomic DNA sequence was constructed. This was used to insert to the 2A-Cre DNA fragment into exon 9 immediately after the stop codon of *Kremen1* gene locus, guided by the gRNA with sequence GTGGGCTTCAGTCACTCACG AGG. One founder mouse was generated, and the correct genomic modification was confirmed by sequencing.

### Laser capture microdissection and RNA sequencing

One-month-old *Nr4a1-eGFP* transgenic mice were euthanized with CO_2_ followed by rapid decapitation. The brains were immediately dissected and preserved at −80 °C. Cryosectioning of the frozen brains was performed at −20 °C, and the sections were mounted onto PAN membrane frame slides (Applied Biosystems, Foster City, CA). The dorsal striatum was determined based on anatomic landmarks such as the corpus callosum, lateral ventricle and nucleus accumbens. Using the ArturusXT microdissection system with fluorescent illumination (Applied Biosystems), the eGFP-positive island-like structures within the dorsal striatum of *Nr4a1*-eGFP transgenic mice were carefully isolated onto LCM Macro Caps (Applied Biosystems) and designated as “patch”, while surrounding tissues of similar size were also isolated onto Macro Caps and designated as “matrix”.

Total RNA extraction and purification were executed from hundreds of caps using the PicoPure Isolation kit (Applied Biosystems), with subsequent genomic DNA clearance facilitated by RNase-free DNase (Qiagen) following the manufacturer’s protocols. Quantification of RNA was performed using a NanoDrop spectrophotometer (ThermoFisher), and RNA integrity was evaluated using the Bioanalyzer RNA 6000 pico assay (Agilent). Only RNA samples exhibiting high integrity were selected for subsequent patch and matrix RNAseq library preparation.

cDNA libraries were prepared from the purified RNA using the TruSeq Stranded Total RNA LT library preparation kit (Illumina) following the manufacturer’s protocol. The quality of the libraries was assessed using the Bioanalyzer DNA 1000 assay (Agilent) before sequencing on an Illumina HiSeq 2000 platform. Fastq les were generated using the standard Illumina pipeline. Transcript abundance, annotated by Ensembl, was quantified using Salmon in a non-alignment-based mode, and gene-level counts were estimated utilizing the Tximport package (Bioconductor). Normalization of counts and subsequent data analysis were conducted following previously established procedures^35^. The accession number of the striatal tissue RNA-seq is PRJNA870469.

### RNA *in situ* hybridization and image analysis

RNA *in situ* hybridization (ACDBio, RNAscope) was used to detect the expression of *Drd1*, *Drd2*, *Kremen1* and *Tshz1* mRNAs in the dorsal striatum of adult C57BL/6J mice. For tissue preparation, mice were anesthetized with CO_2_ and rapidly decapitated. The brains were fresh-frozen on dry ice and stored at −80 °C before sectioning. Striatal sections (12μm) were collected using a cryostat (Leica Biosystems) and stored at −80 °C until processed.

RNAscope was conducted according to the instructions of RNAscope Multiplex Fluorescent Reagent Kit v2 user manual. Probes against *Drd1* (Cat. No. #401901), *Drd2* (Cat. No. #406501), *Kremen1* (Cat. No. #425771) and *Tshz1* (Cat. No. #494291) were applied to brain sections. Fluorescent images were acquired using a laser scanning confocal microscope LSM 780 (Zeiss) with 20× or 40× lens. Bregma coordinates around 1.34mm, 0.92mm, 0.5mm, 0.08mm and −0.34mm were selected for image analysis.

RNAscope images were analyzed using Imaris (v10.0.0, Bitplane, Belfast Northern Ireland, UK). Surfaces for the dorsal and ventral striatum were created using the Allen Brain Atlas as a reference. The spatial patch was defined by outlining *Kremen1*^+^ SPN clusters (a minimum of 5 SPNs), and the density of *Kremen1*^+^ SPNs within a spatial patch is at least 200 cells/mm^2^. Surfaces for individual channels (i.e., *Drd1*, *Drd2*, *Kremen1*, *Tshz1* and DAPI) were created with unique parameters, which were saved and applied to all images within the same batch. Minor adjustments were made in subsequent rounds of RNAscope to improve Quantification accuracy. DAPI surfaces in the striatum were filtered by the mean intensity of *Drd1* (dSPNs) and the mean intensity of *Drd2* (iSPNs). These *Drd1*^+^ and *Drd2*^+^ cells were further filtered by the overlapped volume ratio to *Kremen1* to quantify patch dSPNs and patch iSPNs, respectively. RNAscope analysis with *Tshz1* was conducted in a similar manner, filtering *Drd1*^+^ and *Drd2*^+^ cells by the overlapped volume ratio to the *Tshz1* surface. All data points are presented as the average across striatal sections of both hemisphere for each mouse.

### Immunohistochemistry

Mice were anesthetized with pentobarbital and transcardially perfused with precooled PBS, followed by 4% paraformaldehyde (PFA) solution as described previously^12,36^. Brains were stored in 4% PFA at 4 °C overnight and then transferred to 30% PBS buffered sucrose solution for at least 2 days before sectioning. Series of coronal sections (40μm) were collected using a cryostat and stored at 4°C in PBS. Sections were blocked in 10% normal donkey serum (Sigma-Aldrich) + 0.5% Bovine serum albumin (Sigma-Aldrich) + 0.3% Triton for 1 hr at room temperature (RT). Next, sections were incubated with the primary antibodies over one or two nights at 4 °C. Sections were then washed with PBS (3 × 10 min) and incubated with fluorescent secondary antibodies at RT for 1 hr. In some experiments, sections were further washed twice in PBS, incubated with DAPI (40, 6-diamidino-2-phenylindole, Invitrogen, D1306) (0.5mg/ml in PBS) for 1 min. After washing with PBS (5 min), sections were mounted onto subbed slides, and coverslipped with mounting media (ProLong^®^ Gold Antifade Mountant, Life technology). Images were taken using a laser scanning confocal microscope LSM 780 (Zeiss). The primary antibodies used for immunostaining included rabbit monoclonal anti-TH (Pel-Freez Biologicals, P40101; dilution 1:1000), mouse monoclonal anti-TH (ImmunoStar, 22941; dilution 1:1000), chicken polyclonal anti-TH (Aves Labs, TYH; dilution 1:500), rabbit anti-Mu-opioid receptor (MOR) (ImmunoStar, 24216; dilution 1:3000), chicken polyclonal anti-GFP (Aves Labs, GFP-1020; dilution 1:1000), mouse polyclonal anti-RFP (Rockland, 200-301-379; dilution 1:1000), guinea pig anti-Parvalbumin (Swant, GP72; dilution 1:1000), rabbit anti-Somatostatin-14 (T-4103, Peninsula Laboratories; dilution 1:1000), goat anti-ChAT (AB144P, Millipore; dilution 1:500), rabbit anti-Iba1 (Wako, 019–19741, 1:1000), rabbit anti-GFAP (Abcam, ab7260; dilution 1:2000), goat anti-CD13 (R&D Systems, AF2335; dilution 1:100), and mouse monoclonal anti-GABA_B_ (Abcam, ab55051, dilution 1:500). Appropriate fluorophore-conjugated secondary antibodies (Life Technologies) were used depending on the desired fluorescence colors.

### Viral vectors

The following AAV vectors were purchased from Addgene (Watertown, MA, USA): AAV9-hSyn-DIO-hM4D(Gi)-mCherry (#44362), AAV9-hSyn-DIO-mCherry (#50459), AAV1-EF1a-double floxed-hChR2(H134R)-EYFP (#20298), AAV1-hSyn-hChR2(H134R)-EYFP (#26973), AAV8-hSyn-Con/Fon-hChR2(H134R)-EYFP (#55645), AAV8-hSyn-Con/Fon-EYFP (#55650), AAV1-Ef1a-DIO-EYFP (#27056), and AAV1-syn-FLEX-jGCaMP8s (#162377). AAV2/9-hSyn-DIO-GRAB-rDA3m were produced by BrainVTA (Gaithersburg, MD, USA). AAV9-FLEX-SaCas9-U6-sgGabbr1 was packaged by SignaGen Laboratories (Frederick, MD, USA). AAV9-CMV-NLS-SaCas9-3XHA-sNRPpa-U6-Basal-empty was packaged by Vigene Biosciences (Rockville, MD, USA).

### Construction of pAAV-FLEX-SaCas9-U6-sgGabbr1

The reference sequence of *Gabbr1* gene was retrieved from the UCSC genome browser database (http://genome.ucsc.edu/), and subsequent identification and alignment of exons were performed using the Mouse Genome Informatics (MGI) database. The most 5′ common coding exons were then selected, and the sequence was uploaded to the CRISPOR website (http://crispor.org) for identifying potential sgRNAs and PAM sequences. Three *Gabbr1* sgRNAs were synthesized as short oligos (Euro ns Genomics) with a 5′ CACC-3′ overhang on the forward primer, and a 5′ -AAAC 3′ overhang on the reverse primer, facilitating seamless integration into the pX601-AAV-CMV;NLS-SaCas9-NLS-3xHA-bGHpA;U6;BsaI-sgRNA vector, a gift provided by Dr. Feng Zhang (Addgene plasmid # 61591). Sanger sequencing confirmed the insertion of the *Gabbr1* sgRNA using the primer TAACCACGTGAGGGCCTATTTC.

Mouse Neuro-2a (N2a) cell lines were cultured in Dulbecco’s modified Eagle’s medium (DMEM) supplemented with 10% FBS (HyClone) at 37°C with 5% CO_2_ incubation. Transfection of Cells with the plasmid pX601-AAV-CMV;NLS-SaCas9-NLS-3xHA-bGHpA;U6;*sgGabbr1* vector was performed using Neuro-2a Transfection Kit (Altogen Biosystems), following the manufacturer’s protocol. Cells were then harvested for PCR-based identification of mutations induced by genome editing using the Guide-it Mutation Detection Kit (Cat. No. 631443). The sg*Gabbr1* with the sequence of CACCGCGCCACACTCCACAATCCCAC exhibited high efficiency in genome editing under SaCas9 and was thus selected for further integration into the pAAV-Flex-SaCas9-U6-*sgDaglb* vecgtor^35^. The primers 5′-TAACCACGTGAGGGCCTATTTCCCATGATT and 5′-CGCTCGGTCCGAAAAATCTCGCCAACAAGTT were used to amplify the fragment containing sg*Gabbr1*, which replaced sg*Dalgb* in the plasmid pAAV-Flex-SaCas9-U6-*sgDagl*b at the PmlI and RsrII registration sites. Sanger sequencing confirmed the presence of sg*Gabbr1* in the resulting pAAV-Flex-SaCas9-U6-*sgGabbr1* plasmid.

### Stereotaxic injection and optic fiber implantation

The stereotaxic survival surgery was performed as previously described^35^. All surgery were conducted under aseptic conditions, and body temperature was maintained using a heating pad. Brie y, adult mice (2–4 months old) were anesthetized with isoflurane (1–2%) and head-fixed in a stereotaxic frame (Kopf Instruments). A total volume of 500–700 nL of AAVs was injected unilaterally or bilaterally into the dorsal striatum with two locations per hemisphere (coordinates: 0.5 mm AP to Bregma, ±2.2 mm ML, −2.5 mm DV from dura surface; 1.3 mm AP from Bregma, ±1.8 mm ML, −2.5 mm DV from dura surface) or SN (coordinates: −3.1 mm AP from Bregma, ±1.5 mm ML, −3.9 mm DV from dura surface). The infusion of viruses was controlled by a stereotaxic injector (Stoelting) at a speed of 75 nL/min. After a 5-minute wait following the end of injection, the injector was slowly withdrawn. The scalp was then sutured, and the mice were returned to their home cages. All behavior experiments were performed at least 4 weeks after injection to allow time for full heterologous gene expression. For *Aldh1a1*^CreERT2^ mice, 4-OHT was injected intraperitoneally at a dosage of 10 mg/kg bodyweight for five consecutive days, beginning one week after surgery, to induce *Cre* recombinase expression.

To prepare mice for optogenetics or fiber photometry experiments, we performed a second surgery to implant optic fibers at least 3 weeks after viral injection. For behavioral optogenetics experiments, optical fiber stubs (200 μm core, 0.39 NA, Thorlabs) were bilaterally implanted with the tips positioned over the SNc (−3.1 mm AP from Bregma, ±1.5 mm ML, −3.9 mm to −4.1 mm DV from dura surface) or GPe (−0.3 mm AP from Bregma, ±2.0 mm ML, −3.5 mm from dura surface). The optic fibers were secured in place with a thick layer of radiopaque adhesive cement (C&B METABOND, Parkell). Once dried, Vetbond tissue adhesive (3M) was applied to seal the head incision with adhesive cement.

For simultaneous photometry recordings and optogenetics experiments, optic fiber stubs with a 200 μm core and 0.39 NA (Thorlabs) were unilaterally implanted over the SN (−3.1 mm AP from Bregma, 1.5 mm ML, −3.9 mm to −4.1 mm DV from dura surface) for optogenetic stimulation. In addition, optic fiber stubs with a 200 μm core and 0.5 NA (Plexon) were implanted over dorsolateral striatum (+1.0 mm AP from Bregma, 2.0 mm ML, −2.2 mm to −2.5 mm DV from the dura surface) on the same side for striatal rDA3m photometry recording during optogenetic stimulation.

For the treadmill fiber photometry experiment, optic fiber stubs with a 200 μm core and 0.5 NA (Thorlabs) were unilaterally or bilaterally implanted in the SN (−3.1 mm AP from Bregma, 1.5 mm ML, −4.0 mm to −4.3 mm DV from dura surface) to record the axon calcium signals of patch or matrix dSPNs. The optic fibers were fixed in place using adhesive cement. A titanium metal head-bar (Labeotech) was mounted on top of the adhesive cement rostral to the fiber stubs for head-restraint. The animals were allowed to recover for at least one week after the optic implantation before any optogenetics or fiber photometry experiments.

### Open-field spontaneous locomotion

Locomotion in freely moving mice was measured using video tracing analyses. Mice were habituated for 30 min in the behavioral room, wherein a 20W lamp was shielded in a box and positioned in the dark opposite to the testing area to provide a diffuse light source. For the video tracing test, mice were placed in a 50cm × 50cm grey opaque chamber for 30min. Activity was recorded from overhead by a digital camera at a frame rate of 15 Hz. EthoVision XT software (Noldus) was used to track the mice and analyze the video for velocity, time, and distance travelled.

### Chemogenetic manipulation

JHU37160-dihydrochloride (Hello Bio) was dissolved in water to achieve a stock concentration of 0.3 mg/mL and stored in small aliquots at −20 °C. Prior to each use, the working solution was freshly prepared by diluting the stock 10-fold with 0.9% saline. Mice received 0.3mg/kg bodyweight dosage via intraperitoneal injection 30 minutes before behavioral tests.

### Optogenetic stimulation

A LED light source (PlexBright, Plexon) was connected to a multimodal optic fiber patch cable (200 μm core, 0.39 NA, Plexon) through ceramic sleeves (Thorlabs) to the ferrule of the optic fiber stubs previously implanted in the mouse. Light power was calibrated using a power meter (PM100D, Thorlabs) to achieve the desired output measured at the tip of optic fiber. For ChR2 experiments, photo stimulation consisted of 5ms-width blue light pluses (465nm, 3mW) at different frequency or constant blue light (465nm, 0.25mW). Light pulses were generated by a TTL pulse generator (OPTG-4, Doric Lenses).

#### Optogenetics with open field

Mice were habituated in the testing room 30min before testing, and the apparatus was cleaned with 50% ethanol between animals. Mice were placed in a 50cm × 50cm clear chamber and their activities were captured by top-view and side-view cameras (Logitech). After a 3min exploration period, mice received either 3min ON and 3min OFF bilateral stimulation or 10s ON and 1min OFF bilateral stimulation. Video and LED light TTL were acquired and synchronized with Synapse software (Tucker-Davis Techonologies). Distanced traveled and velocity during the acquisition period were calculated using EthoVision XT software. Ambulation bouts were scored as periods of movement > 2cm/s lasting for > 0.5s and separated by > 0.5s. Immobility bouts were scored as periods of < 2% pixel change lasting for > 0.5s and separated by > 0.5s.

#### Optogenetics with fiber photometry

Mice were allowed to freely move in an open field chamber and received photostimulation of 2s, 5s, or 15s for a total of approximately 10 trials for each period setting, with intervals of at least 45s between trials. The rDA3m signals were recorded simultaneously with videotaping (see “Fiber Photometry section). We established the baseline by determining the average ΔF/F value in the 2 s preceding stimulation. The reduction in dopamine amplitude was computed by subtracting the average ΔF/F value in the 0.2 s prior to stimulus offset from the baseline. For *Calb1*^IRES2-Cre^ mice subjected to matrix dSPNs stimulation, the dopamine peak was determined by subtracting the average ΔF/F during a 0.1s interval between 0.35 to 0.45s after stimulus onset from the baseline. Additionally, post-stimulus dopamine change amplitude was calculated by subtracting the average ΔF/F during a 5-sec interval between 3 to 8s after stimulus offset from the baseline.

### Fiber Photometry

Dopamine sensor or GCaMP8s fluorescence was measured using a locked-in amplifier system (Tucker-Davis Technologies, Model RZ10X with Synapse software). The photometry recordings were conducted with either green (GCaMP8s) or red (rDA3m) fluorescence. For GCaMP8s recordings, the blue LED (465nm) was sinusoidally modulated at 330 Hz, and the UV LED (405nm) was modulated at 211 Hz as an isosbestic control channel. For rDA3m recordings, the green LED (560nm) was modulated at 410 Hz, and the UV LED was modulated at 211 Hz. The peak intensity of each LED was calibrated to 20–60 μW, measured at the distal end of the patch cable. Light emissions were filtered through a 6-port fluorescence mixing cube (Doric Lens) before being coupled to an optic patch cable (200μm core, 0.5 NA), affixed to the implanted optic fiber in each mouse. The emitted fluorescent signals were collected by integrated photosensors in RZ10X real-time signal processor equipped with lock-in amplifier. Transistor-transistor logic (TTL) signals were employed to timestamp onset times for each event of interest (e.g., stimulation onset, locomotion onset and offset), which were detected via the RZ10X in the Synapse software. Fiber photometry data was analyzed using custom MATLAB code. Demodulated 465nm, 560nm, and 405nm recording traces were recorded at a sampling rate of 1k Hz.

For the analysis of rDA3m signals during optogenetic stimulation, to capture the large offset change caused by optogenetic stimulation, the demodulated photometry trace of rDA3m was normalized to compute ΔF/F. F was estimated as a referenced rDA3m signal, where a RANSAC ordinary least square linear regression was performed between the demodulated UV isosbestic reference signal and demodulated rDA3m signal to transform the reference signal to account for the differences in gain and offset between the two signals, as well as possible motion artefacts and long-term photo bleaching effects. Then the referenced rDA3m signal (F) was subtracted from the demodulated rDA3m signal to compute ΔF.

To analyze the modulation of GCaMP8s activity change with locomotor behavior, photometry signal normalization followed a published study^37^, using custom Matlab code. Demodulated photometry traces of both GCaMP8s and UV isosbestic reference channels were pre-normalized by first computing ΔF/F0. F0 was estimated by calculating the 10th percentile of the raw photometry amplitude using a 5-sec sliding window to account for slow, correlated fluorescence changes, including photobleaching in both channels. ΔF was calculated as raw photometry amplitude subtracted its respective F0. Both channels were initially normalized with this procedure. An additional referencing procedure was performed to remove the effects of motion or mechanical artefacts from analysis. For this referencing procedure, the ΔF/F0 of the UV reference signal was low-pass filtered with a second-order Butterworth filter with a 3 Hz corner frequency. Then a RANSAC ordinary least squares regression between the filtered reference signal and normalized GCaMP8s signal was used to transform the reference signal to account for the differences in gain and offset between the two signals. Lastly, the transformed references trace was subtracted from the normalized GCaMP8s trace as the final ΔF/F. The transformed reference trace was then used as UV ΔF/F. Only the experiments where the maximum percentage of DF/F exceeded 1.5 and the GCaMP8s and the UV reference correlation was below 0.6 were included for further analysis. Z-score of ΔF/F was calculated with mean and standard deviation of the entire recording session.

### Head-fixed voluntary treadmill with fiber photometry

The head-fixed locomotion was achieved with a compact low friction manually driven treadmill, originally designed by Janelia Research Campus of the HHMI, purchased from LABmaker (Berlin, Germany). Te on belt movement was tracked by a rotary encoder, which sends 0–3.3V analog output of speed and direction to an ADC port at TDT RZ10x to synchronize with photometry recordings. The speed was calibrated with 10 cm/s at 2.5V and sampled at 1k Hz. The head fixture was achieved through head fixation system along with an implantable titanium head bar from Labeotech (Montreal, Canada). Mice started treadmill habituation training one week after recovering from head-bar implant surgery to get used to walking on treadmill while head was fixed. Each habituation session lasted 20min and repeated at most twice a day for 3–4 days until mice were able to spontaneously initiate at least 20 walk bouts during a 20min session. After habituation training, a 30min fiber photometry recording session was performed for each individual mouse, while they walked on the treadmill with head fixation.

Movement versus rest time bins were defined with a 0.25 cm/s threshold on the velocity trace. Isolated movement periods with duration shorter than 0.5s or movement periods with average velocity smaller than 0.5cm/s were excluded for analysis. Because mice tended to walk slower on the treadmill, time bins were considered as rest periods only if they lasted longer than 0.8s. A movement bout initiation time was defined as velocity cross the threshold at the end of the rest period, and a movement termination time was defined as the time velocity fell below the threshold followed by a rest period. Timestamp of maximum value of z-scored GCaMP activity during 1s before and 1s after locomotion onset or offset was calculate the time of maximum activity for the respective events. Activity slope during locomotion offset was computed as the coefficient of a least-square linear regression t for the z-scored GCaMP activity during the interval of 0.5s before and after locomotion offset.

### Statistics

Data were analyzed by Prism 9 software (Graphpad) and custom code written in MATLAB (MathWorks). All statistical details for each experiment can be found in the corresponding figure legends. Data were presented as means and standard errors of the mean (SEM) or median and minimum or maximum. We assessed the statistical significance using parametric t-test and two-way analysis of variance (ANOVA). Significance was considered for test statistics with a (*) p-value of < 0.05, (**) p-value of < 0.01, (***) p-value of < 0.001.

## Discussion

In this study we challenge the canonical model suggesting that striatonigral dSPNs promote locomotion and demonstrate that this model perhaps only applies to the matrix striatonigral dSPNs, while the patch striatonigral dSPNs play an opposing role by suppressing locomotion. This inhibitory function of patch dSPNs is implicated in the rise of neuronal activity during the offset of self-paced movement and is causally validated by optogenetic activation. Furthermore, we showed the critical involvement of GABA-B receptors in ALDH1A1^+^ nigrostriatal DANs during patch dSPN-induced locomotion inhibition.

The dSPNs and iSPNs are subdivided into patch and matrix compartments based on neurochemical markers and their input-output connectivity;^3^ however, it remains challenging to ambiguously define the patch compartments. Conventional patch markers, including MOR1, substance P and enkephalin, can only identify a portion of patch compartments along the rostral to caudal axis of the striatum^22^. Specifically, MOR1 marks the patch in the rostral and intermediate striatum, while substance P and enkephalin label the patch in the intermediate and caudal striatum^22^. In contrast, *Kremen1* marks the patch structures within the entire dorsal striatum, including the dorsolateral striatum, a region heavily involved in motor control and learning^23^. Additionally, *Kremen1* marks both *Drd1*-and *Drd2*-expressing SPNs in the same ratio as the general SPN population, while MOR1 tends to label more *Drd1*-expressing dSPNs^24^. In addition to MOR1, *Pdyn* and *Tshz1* are also preferentially mark the patch dSPNs^9, 10^. Notably, there is less than 3% overlap between the *Pdyn*^+^ and *Tshz1*^+^ Drd1-expressing dSPNs^25^, suggesting heterogeneity among patch SPNs based on different gene expression pro les. Furthermore, substantially more *Pdyn*^+^ and *Tshz1*^+^ SPNs are distributed in the matrix compartments compared to the *Kremen1*^+^ SPNs. Therefore, *Kremen1* serves as a useful marker for a distinctive population of patch SPNs, which unbiasedly labels both dSPNs and iSPNs selectively in the patch compartments.

The *Pdyn*-positive (*Pdyn*^+^) dSPNs were suggested to promote locomotion, while the *Tshz1*-positive (*Tshz1*^+^) ones were claimed to suppress locomotion in a place-preference test^25^. Since patch dSPNs have been implicated in discriminating valence during decision-making, changes in movement velocity by patch dSPN activation in valence context (such as place preference test) may reflect alterations in active avoidance or approach choices rather than locomotion itself^11^. Moreover, the roles of SNr-projecting dSPNs were not specifically examined in the aforementioned study^25^, considering the GPi-projecting dSPNs may originate from a distinct population^26^. As *Tshz1*^+^ patch dSPNs have also been associated with locomotion suppression^25^, we conducted a comparison of *Kremen1*^+^ and *Tshz1*^+^ Drd1-expressing neurons in the dorsal striatum. Our findings reveal that *Kremen1* and *Tshz1* mark two largely different subtypes of patch dSPNs in the dorsal striatum. While we demonstrate that SNr-projecting *Kremen1*^+^ dSPNs inhibit ambulatory locomotion, whether SNr-projecting *Tshz1*^+^ SPNs serve a similar function remains unclear^25^. Given the significant diversity among patch SPNs based on molecular markers and connectivity, future studies should explicitly identify subtypes using genetic markers and projection targets.

Both patch and matrix dSPN activity showed a similar transient increase at the onset of locomotion, but started to diverge as movement ceased, with matrix dSPN exhibiting decreased activity while patch dSPN transiently increased activity. The activity pattern of patch and matrix dSPNs during locomotion mirrors previous findings of synergistic concurrent activation of opposing dSPNs and iSPNs during movement initiation^24^ with their activity decorrelating as movement progresses^27^. Furthermore, manipulating these two populations of dSPNs causally produced opposing locomotor effects. These findings highlight the presence of opposing motifs within the dSPN population, one originating from the patch and the other from matrix, akin to the dichotomous organization of dSPN and iSPN in the broader dorsal striatum. Our results suggest that patch dSPN activity may facilitate state transition from movement to quiescent. In support of this hypothesis, patch dSPN activity began to ramp hundreds of milliseconds before movement termination, and optogenetic activation of patch dSPNs produced state-dependent locomotor effect, shortening movement bout duration while increasing instance of immobility. State transition regulation has been identified as a fundamental principle of basal ganglia motor control^28^. In addition to receive sensorimotor inputs, patch dSPNs receive more limbic inputs than matrix dSPNs^29^ and have robust direct connections with midbrain DANs, suggesting their role in integrating external sensorimotor information with implicit motivation to regulate motor state transitions. Conversely, we found that patch iSPNs exerted a positive, albeit less pronounced, effect on ambulatory movement upon activation compared to patch dSPNs. This aligns with previous findings suggesting that striatal iSPNs apply inhibitory motor control through their GABAergic axon collaterals to surrounding dSPNs, while the majority of striatopallidal inputs drive negative reinforcement learning^30^. The synaptic strength of iSPN-dSPN connections within the patch remains unclear. Given the relatively smaller number of patch iSPNs, their collaterals within the patch may not be as robust. Further exploration is warranted to elucidate the function of patch iSPNs.

ALDH1A1^+^ nigrostriatal DANs receive the most monosynaptic inputs from the dorsal striatum^12^. Furthermore, these neurons appear to form reciprocal innervation with dSPNs in the dorsal regions of dorsal striatum^12^. This reciprocal connection between ALDH1A1^+^ DANs and dSPNs may constitute a feedback loop for timely regulation of the dopamine release and SPN activity in motor control and learning. While both patch and matrix dSPNs innervate ALDH1A1^+^ DANs^12^, the axon terminals of patch dSPNs are the primary presynaptic components in this so-called striosome-dendron bouquet structure with the dendrites of ALDH1A1^+^ nigrostriatal DANs^14, 15^. This arrangement potentially plays a pivotal role in regulating dopamine release in both dorsal striatum and SNr. Accordingly, brain slice recordings indicate that patch dSPNs deliver stronger inhibitory inputs to the DANs and could induce rebound ring in ALDH1A1^+^ nigrostriatal DANs compared to matrix dSPNs and the other inhibitory inputs^14, 16^. Consistent with these in vitro studies, our in vivo recordings reveal a more pronounced inhibition of dopamine release upon stimulating patch dSPNs compared to the matrix dSPNs, with a rebound dopamine release observed upon the cessation of patch dSPN stimulation. Supporting the functional significance of the connection between patch dSPNs and ALDH1A1^+^ nigrostriatal DANs, genetic deletion of GABA-B receptors in ALDH1A1^+^ nigrostriatal DANs completely abolished the locomotion-suppressing effects mediated by patch dSPNs. This synaptic coupling might dynamically regulate the dopamine supply in both the dorsal striatum and SNr, thereby modulating various motor activities.

Notably, the activation of matrix dSPNs initially triggered a transient increase in dopamine release before a subsequent reduction occurred. Considering that matrix dSPNs project to inhibitory parvalbumin neurons (PVNs) in the SNr and PVNs provide direct and tonic inhibitory inputs to DANs^31–34^, it’s plausible that matrix dSPNs initially disinhibit DANs via PVNs, resulting in an early increase in DAN activity and dopamine release. The prevalence of this transient increase of dopamine release within the first two seconds of matrix dSPN stimulation suggests that the primary effect of matrix dSPN activation on DANs is disinhibition or excitation. Therefore, the distinct modulation of DAN activity by patch and matrix dSPNs may underlie their differentia influence on motor control.

## Methods

### Mouse work

All mouse studies were conducted in accordance with the guidelines approved by the Institutional Animal Care and Use Committees (IACUC) of the Intramural Research Program of the National Institute on Aging (NIA), NIH. All mouse lines were maintained as heterozygotes in a C57BL/6J background. The *Kremen1*^2A − Cre^ KI mice were generated by Shanghai Model Organisms Inc. (Shanghai, China). The Aldh1a1^CreERT2^ KI mice were generated as previously described^12^. The Nr4a1–eGFP (Stock No: 036737-UCD) transgenic mice were obtained from Mutant Mouse Resource & Research Centers (MMRRC). The Calb1^IRES2 − Cre^ mice (Stock No: 028523) and Ai14 (Stock No: 007908) were obtained from the Jackson laboratory. The A2a^Flp^ KI mice were generated by the Rodent Transgenic Core of National Institute of Mental Health (NIMH). Both females and males were used for all experiments. Mice used for viral injections were between 2 and 4 months of age. The mice were housed in a 12-hour-light/12-hour-dark cycle in groups of 2–5 animals and had ad libitum water and a regular diet. All the behavioral tasks were performed during the light cycle. Littermates were randomly assigned to different groups prior to experiments.

#### Generation of *Kremen1*^2A − Cre^ KI mice

The *Kremen1*^2A − Cre^ KI mice were generated using the CRIPSR/Cas9 approach in the C57BL/6J strain by Shanghai Model Organisms Inc. A donor plasmid containing the 2A ribosome skipping sequence, Cre DNA recombinase sequence, and flanking *Kremen1* mouse genomic DNA sequence was constructed. This was used to insert to the 2A-Cre DNA fragment into exon 9 immediately after the stop codon of *Kremen1* gene locus, guided by the gRNA with sequence GTGGGCTTCAGTCACTCACG AGG. One founder mouse was generated, and the correct genomic modification was confirmed by sequencing.

### Laser capture microdissection and RNA sequencing

One-month-old Nr4a1-eGFP transgenic mice were euthanized with CO_2_ followed by rapid decapitation. The brains were immediately dissected and preserved at − 80°C. Cryosectioning of the frozen brains was performed at − 20°C, and the sections were mounted onto PAN membrane frame slides (Applied Biosystems, Foster City, CA). The dorsal striatum was determined based on anatomic landmarks such as the corpus callosum, lateral ventricle and nucleus accumbens. Using the ArturusXT microdissection system with fluorescent illumination (Applied Biosystems), the eGFP-positive island-like structures within the dorsal striatum of Nr4a1-eGFP transgenic mice were carefully isolated onto LCM Macro Caps (Applied Biosystems) and designated as “patch”, while surrounding tissues of similar size were also isolated onto Macro Caps and designated as “matrix”.

Total RNA extraction and purification were executed from hundreds of caps using the PicoPure Isolation kit (Applied Biosystems), with subsequent genomic DNA clearance facilitated by RNase-free DNase (Qiagen) following the manufacturer’s protocols. Quantification of RNA was performed using a NanoDrop spectrophotometer (ThermoFisher), and RNA integrity was evaluated using the Bioanalyzer RNA 6000 pico assay (Agilent). Only RNA samples exhibiting high integrity were selected for subsequent patch and matrix RNAseq library preparation.

cDNA libraries were prepared from the purified RNA using the TruSeq Stranded Total RNA LT library preparation kit (Illumina) following the manufacturer’s protocol. The quality of the libraries was assessed using the Bioanalyzer DNA 1000 assay (Agilent) before sequencing on an Illumina HiSeq 2000 platform. Fastq les were generated using the standard Illumina pipeline. Transcript abundance, annotated by Ensembl, was quantified using Salmon in a non-alignment-based mode, and gene-level counts were estimated utilizing the Tximport package (Bioconductor). Normalization of counts and subsequent data analysis were conducted following previously established procedures^35^. The accession number of the striatal tissue RNA-seq is PRJNA870469.

#### RNA in situ hybridization and image analysis

RNA in situ hybridization (ACDBio, RNAscope) was used to detect the expression of *Drd1*, *Drd2*, *Kremen1* and *Tshz1* mRNAs in the dorsal striatum of adult C57BL/6J mice. For tissue preparation, mice were anesthetized with CO_2_ and rapidly decapitated. The brains were fresh-frozen on dry ice and stored at −80°C before sectioning. Striatal sections (12μm) were collected using a cryostat (Leica Biosystems) and stored at −80°C until processed.

RNAscope was conducted according to the instructions of RNAscope Multiplex Fluorescent Reagent Kit v2 user manual. Probes against *Drd1* (Cat. No. #401901), *Drd2* (Cat. No. #406501), *Kremen1* (Cat. No. #425771) and *Tshz1* (Cat. No. #494291) were applied to brain sections. Fluorescent images were acquired using a laser scanning confocal microscope LSM 780 (Zeiss) with 20× or 40× lens. Bregma coordinates around 1.34mm, 0.92mm, 0.5mm, 0.08mm and − 0.34mm were selected for image analysis.

RNAscope images were analyzed using Imaris (v10.0.0, Bitplane, Belfast Northern Ireland, UK). Surfaces for the dorsal and ventral striatum were created using the Allen Brain Atlas as a reference. The spatial patch was defined by outlining *Kremen1*^+^ SPN clusters (a minimum of 5 SPNs), and the density of *Kremen1*^+^ SPNs within a spatial patch is at least 200 cells/mm^2^. Surfaces for individual channels (i.e., *Drd1*, *Drd2*, *Kremen1*, *Tshz1* and DAPI) were created with unique parameters, which were saved and applied to all images within the same batch. Minor adjustments were made in subsequent rounds of RNAscope to improve Quantification accuracy. DAPI surfaces in the striatum were filtered by the mean intensity of *Drd1* (dSPNs) and the mean intensity of *Drd2* (iSPNs). These *Drd1*^+^ and *Drd2*^+^ cells were further filtered by the overlapped volume ratio to *Kremen1* to quantify patch dSPNs and patch iSPNs, respectively. RNAscope analysis with *Tshz1* was conducted in a similar manner, filtering *Drd1*^+^ and *Kremen1*^+^ cells by the overlapped volume ratio to the *Tshz1* surface. All data points are presented as the average across striatal sections of both hemisphere for each mouse.

### Immunohistochemistry

Mice were anesthetized with pentobarbital and transcardially perfused with precooled PBS, followed by 4% paraformaldehyde (PFA) solution as described previously^12, 36^. Brains were stored in 4% PFA at 4°C overnight and then transferred to 30% PBS buffered sucrose solution for at least 2 days before sectioning. Series of coronal sections (40μm) were collected using a cryostat and stored at 4°C in PBS. Sections were blocked in 10% normal donkey serum (Sigma-Aldrich) + 0.5% Bovine serum albumin (Sigma-Aldrich) + 0.3% Triton for 1 hr at room temperature (RT). Next, sections were incubated with the primary antibodies over one or two nights at 4°C. Sections were then washed with PBS (3 × 10 min) and incubated with fluorescent secondary antibodies at RT for 1 hr. In some experiments, sections were further washed twice in PBS, incubated with DAPI (40, 6-diamidino-2-phenylindole, Invitrogen, D1306) (0.5mg/ml in PBS) for 1 min. After washing with PBS (5 min), sections were mounted onto subbed slides, and coverslipped with mounting media (ProLong^®^ Gold Antifade Mountant, Life technology). Images were taken using a laser scanning confocal microscope LSM 780 (Zeiss). The primary antibodies used for immunostaining included rabbit monoclonal anti-TH (Pel-Freez Biologicals, P40101; dilution 1:1000), mouse monoclonal anti-TH (ImmunoStar, 22941; dilution 1:1000), chicken polyclonal anti-TH (Aves Labs, TYH; dilution 1:500), rabbit anti-Mu-opioid receptor (MOR) (ImmunoStar, 24216; dilution 1:3000), chicken polyclonal anti-GFP (Aves Labs, GFP-1020; dilution 1:1000), mouse polyclonal anti-RFP (Rockland, 200-301-379; dilution 1:1000), guinea pig anti-Parvalbumin (Swant, GP72; dilution 1:1000), rabbit anti-Somatostatin-14 (T-4103, Peninsula Laboratories; dilution 1:1000), goat anti-ChAT (AB144P, Millipore; dilution 1:500), rabbit anti-Iba1 (Wako, 019–19741, 1:1000), rabbit anti-GFAP (Abcam, ab7260; dilution 1:2000), goat anti-CD13 (R&D Systems, AF2335; dilution 1:100), and mouse monoclonal anti-GABA_B_ (Abcam, ab55051, dilution 1:500). Appropriate fluorophore-conjugated secondary antibodies (Life Technologies) were used depending on the desired fluorescence colors.

### Viral vectors

The following AAV vectors were purchased from Addgene (Watertown, MA, USA): AAV9-hSyn-DIOhM4D(Gi)-mCherry (#44362), AAV9-hSyn-DIO-mCherry (#50459), AAV1-EF1a-double floxed-hChR2(H134R)-EYFP (#20298), AAV1-hSyn-hChR2(H134R)-EYFP (#26973), AAV8-hSyn-Con/Fon-hChR2(H134R)-EYFP (#55645), AAV8-hSyn-Con/Fon-EYFP (#55650), AAV1-Ef1a-DIO-EYFP (#27056), and AAV1-syn-FLEX-jGCaMP8s (#162377). AAV2/9-hSyn-DIO-GRAB-rDA3m were produced by BrainVTA (Gaithersburg, MD, USA). AAV9-FLEX-SaCas9-U6-sgGabbr1 was packaged by SignaGen Laboratories (Frederick, MD, USA). AAV9-CMV-NLS-SaCas9-3XHA-sNRPpa-U6-Basal-empty was packaged by Vigene Biosciences (Rockville, MD, USA).

#### Construction of pAAV-FLEX-SaCas9-U6-sg Gabbr1

The reference sequence of Gabbr1 gene was retrieved from the UCSC genome browser database (http://genome.ucsc.edu/), and subsequent identification and alignment of exons were performed using the Mouse Genome Informatics (MGI) database. The most 5′ common coding exons were then selected, and the sequence was uploaded to the CRISPOR website (http://crispor.org) for identifying potential sgRNAs and PAM sequences. Three Gabbr1 sgRNAs were synthesized as short oligos (Euro ns Genomics) with a 5′ CACC-3′ overhang on the forward primer, and a 5′ -AAAC 3′ overhang on the reverse primer, facilitating seamless integration into the pX601-AAV-CMV;NLS-SaCas9-NLS-3xHAbGHpA;U6;BsaI-sgRNA vector, a gift provided by Dr. Feng Zhang (Addgene plasmid # 61591). Sanger sequencing confirmed the insertion of the Gabbr1 sgRNA using the primer TAACCACGTGAGGGCCTATTTC.

Mouse Neuro-2a (N2a) cell lines were cultured in Dulbecco’s modified Eagle’s medium (DMEM) supplemented with 10% FBS (HyClone) at 37°C with 5% CO_2_ incubation. Transfection of Cells with the plasmid pX601-AAV-CMV;NLS-SaCas9-NLS-3xHA-bGHpA;U6;sgGabbr1 vector was performed using Neuro-2a Transfection Kit (Altogen Biosystems), following the manufacturer’s protocol. Cells were then harvested for PCR-based identification of mutations induced by genome editing using the Guide-it Mutation Detection Kit (Cat. No. 631443). The sgGabbr1 with the sequence of CACCGCGCCACACTCCACAATCCCAC exhibited high efficiency in genome editing under SaCas9 and was thus selected for further integration into the pAAV-Flex-SaCas9-U6-sgDaglb vecgtor^35^. The primers 5′- TAACCACGTGAGGGCCTATTTCCCATGATT and 5′-CGCTCGGTCCGAAAAATCTCGCCAACAAGTT were used to amplify the fragment containing sg*Gabbr1*, which replaced sg*Dalgb* in the plasmid pAAV-Flex-SaCas9-U6-*sgDaglb* at the PmlI and *RsrII* registration sites. Sanger sequencing confirmed the presence of sg*Gabbr1* in the resulting pAAV-Flex-SaCas9-U6-*sgGabbr1* plasmid.

### Stereotaxic injection and optic fiber implantation

The stereotaxic survival surgery was performed as previously described^35^. All surgery were conducted under aseptic conditions, and body temperature was maintained using a heating pad. Brie y, adult mice (2–4 months old) were anesthetized with isoflurane (1–2%) and head-fixed in a stereotaxic frame (Kopf Instruments). A total volume of 500–700 nL of AAVs was injected unilaterally or bilaterally into the dorsal striatum with two locations per hemisphere (coordinates: 0.5 mm AP to Bregma, ± 2.2 mm ML, − 2.5 mm DV from dura surface; 1.3 mm AP from Bregma, ± 1.8 mm ML, − 2.5 mm DV from dura surface) or SN (coordinates: −3.1 mm AP from Bregma, ± 1.5 mm ML, − 3.9 mm DV from dura surface). The infusion of viruses was controlled by a stereotaxic injector (Stoelting) at a speed of 75 nL/min. After a 5-minute wait following the end of injection, the injector was slowly withdrawn. The scalp was then sutured, and the mice were returned to their home cages. All behavior experiments were performed at least 4 weeks after injection to allow time for full heterologous gene expression. For Aldh1a1^CreERT2^ mice, 4-OHT was injected intraperitoneally at a dosage of 10 mg/kg bodyweight for five consecutive days, beginning one week after surgery, to induce Cre recombinase expression.

To prepare mice for optogenetics or fiber photometry experiments, we performed a second surgery to implant optic fibers at least 3 weeks after viral injection. For behavioral optogenetics experiments, optical fiber stubs (200 μm core, 0.39 NA, Thorlabs) were bilaterally implanted with the tips positioned over the SNc (− 3.1 mm AP from Bregma, ± 1.5 mm ML, − 3.9 mm to − 4.1 mm DV from dura surface) or GPe (− 0.3 mm AP from Bregma, ± 2.0 mm ML, − 3.5 mm from dura surface). The optic fibers were secured in place with a thick layer of radiopaque adhesive cement (C&B METABOND, Parkell). Once dried, Vetbond tissue adhesive (3M) was applied to seal the head incision with adhesive cement.

For simultaneous photometry recordings and optogenetics experiments, optic fiber stubs with a 200 μm core and 0.39 NA (Thorlabs) were unilaterally implanted over the SN (− 3.1 mm AP from Bregma, 1.5 mm ML, − 3.9 mm to − 4.1 mm DV from dura surface) for optogenetic stimulation. In addition, optic fiber stubs with a 200 μm core and 0.5 NA (Plexon) were implanted over dorsolateral striatum (+ 1.0 mm AP from Bregma, 2.0 mm ML, − 2.2 mm to − 2.5 mm DV from the dura surface) on the same side for striatal rDA3m photometry recording during optogenetic stimulation.

For the treadmill fiber photometry experiment, optic fiber stubs with a 200 μm core and 0.5 NA (Thorlabs) were unilaterally or bilaterally implanted in the SN (− 3.1 mm AP from Bregma, 1.5 mm ML, − 4.0 mm to − 4.3 mm DV from dura surface) to record the axon calcium signals of patch or matrix dSPNs. The optic fibers were fixed in place using adhesive cement. A titanium metal head-bar (Labeotech) was mounted on top of the adhesive cement rostral to the fiber stubs for head-restraint. The animals were allowed to recover for at least one week after the optic implantation before any optogenetics or fiber photometry experiments.

### Open-field spontaneous locomotion

Locomotion in freely moving mice was measured using video tracing analyses. Mice were habituated for 30 min in the behavioral room, wherein a 20W lamp was shielded in a box and positioned in the dark opposite to the testing area to provide a diffuse light source. For the video tracing test, mice were placed in a 50cm × 50cm grey opaque chamber for 30min. Activity was recorded from overhead by a digital camera at a frame rate of 15 Hz. EthoVision XT software (Noldus) was used to track the mice and analyze the video for velocity, time, and distance travelled.

### Chemogenetic manipulation

JHU37160-dihydrochloride (Hello Bio) was dissolved in water to achieve a stock concentration of 0.3 mg/mL and stored in small aliquots at −20°C. Prior to each use, the working solution was freshly prepared by diluting the stock 10-fold with 0.9% saline. Mice received 0.3mg/kg bodyweight dosage via intraperitoneal injection 30 minutes before behavioral tests.

### Optogenetic stimulation

A LED light source (PlexBright, Plexon) was connected to a multimodal optic fiber patch cable (200 μm core, 0.39 NA, Plexon) through ceramic sleeves (Thorlabs) to the ferrule of the optic fiber stubs previously implanted in the mouse. Light power was calibrated using a power meter (PM100D, Thorlabs) to achieve the desired output measured at the tip of optic fiber. For ChR2 experiments, photo stimulation consisted of 5ms-width blue light pluses (465nm, 3mW) at different frequency or constant blue light (465nm, 0.25mW). Light pulses were generated by a TTL pulse generator (OPTG-4, Doric Lenses).

### Optogenetics with open field

Mice were habituated in the testing room 30min before testing, and the apparatus was cleaned with 50% ethanol between animals. Mice were placed in a 50cm × 50cm clear chamber and their activities were captured by top-view and side-view cameras (Logitech). After a 3min exploration period, mice received either 3min ON and 3min OFF bilateral stimulation or 10s ON and 1min OFF bilateral stimulation. Video and LED light TTL were acquired and synchronized with Synapse software (Tucker-Davis Technologies). Distanced traveled and velocity during the acquisition period were calculated using EthoVision XT software. Ambulation bouts were scored as periods of movement > 2cm/s lasting for > 0.5s and separated by > 0.5s. Immobility bouts were scored as periods of < 2% pixel change lasting for > 0.5s and separated by > 0.5s. For behavioral-state dependent effects of optogenetic stimulation experiment, mice received 10s ON and 1 min OFF bilateral 20Hz stimulation. Ambulation state was determined by movement velocity > 2cm/s in the entire 0.5 second immediately preceded either stimulation onset or stimulation offset. Quiescence (includes small movement) state was determined by movement velocity < 1.5cm/s in the entire 0.5 second immediately preceded either stimulation onset or stimulation offset. Due to kinematics of stimulation on behavior, Quantification for velocity change used 1 sec interval instead.

### Optogenetics with fiber photometry

Mice were allowed to freely move in an open field chamber and received photostimulation of 2s, 5s, or 15s for a total of approximately 10 trials for each period setting, with intervals of at least 45s between trials. The rDA3m signals were recorded simultaneously with videotaping (see “Fiber Photometry section). We established the baseline by determining the average ΔF/F value in the 2 s preceding stimulation. The reduction in dopamine amplitude was computed by subtracting the average ΔF/F value in the 0.2 s prior to stimulus offset from the baseline. For Calb1^IRES2 − Cre^ mice subjected to matrix dSPNs stimulation, the dopamine peak was determined by subtracting the average ΔF/F during a 0.1s interval between 0.35 to 0.45s after stimulus onset from the baseline. Additionally, post-stimulus dopamine change amplitude was calculated by subtracting the average ΔF/F during a 5-sec interval between 3 to 8s after stimulus offset from the baseline.

### Fiber Photometry

Dopamine sensor or GCaMP8s fluorescence was measured using a locked-in amplifier system (Tucker-Davis Technologies, Model RZ10X with Synapse software). The photometry recordings were conducted with either green (GCaMP8s) or red (rDA3m) fluorescence. For GCaMP8s recordings, the blue LED (465nm) was sinusoidally modulated at 330 Hz, and the UV LED (405nm) was modulated at 211 Hz as an isosbestic control channel. For rDA3m recordings, the green LED (560nm) was modulated at 410 Hz, and the UV LED was modulated at 211 Hz. The peak intensity of each LED was calibrated to 20–60 μW, measured at the distal end of the patch cable. Light emissions were filtered through a 6-port fluorescence mixing cube (Doric Lens) before being coupled to an optic patch cable (200μm core, 0.5 NA), affixed to the implanted optic fiber in each mouse. The emitted fluorescent signals were collected by integrated photosensors in RZ10X real-time signal processor equipped with lock-in amplifier. Transistor-transistor logic (TTL) signals were employed to timestamp onset times for each event of interest (e.g., stimulation onset, locomotion onset and offset), which were detected via the RZ10X in the Synapse software. Fiber photometry data was analyzed using custom MATLAB code. Demodulated 465nm, 560nm, and 405nm recording traces were recorded at a sampling rate of 1k Hz.

For the analysis of rDA3m signals during optogenetic stimulation, to capture the large offset change caused by optogenetic stimulation, the demodulated photometry trace of rDA3m was normalized to compute ΔF/F. F was estimated as a referenced rDA3m signal, where a RANSAC ordinary least square linear regression was performed between the demodulated UV isosbestic reference signal and demodulated rDA3m signal to transform the reference signal to account for the differences in gain and offset between the two signals, as well as possible motion artefacts and long-term photo bleaching effects. Then the referenced rDA3m signal (F) was subtracted from the demodulated rDA3m signal to compute ΔF.

To analyze the modulation of GCaMP8s activity change with locomotor behavior, photometry signal normalization followed a published study^37^, using custom Matlab code. Demodulated photometry traces of both GCaMP8s and UV isosbestic reference channels were pre-normalized by first computing ΔF/F0. F0 was estimated by calculating the 10th percentile of the raw photometry amplitude using a 5-sec sliding window to account for slow, correlated fluorescence changes, including photobleaching in both channels. ΔF was calculated as raw photometry amplitude subtracted its respective F0. Both channels were initially normalized with this procedure. An additional referencing procedure was performed to remove the effects of motion or mechanical artefacts from analysis. For this referencing procedure, the ΔF/F0 of the UV reference signal was low-pass filtered with a second-order Butterworth filter with a 3 Hz corner frequency. Then a RANSAC ordinary least squares regression between the filtered reference signal and normalized GCaMP8s signal was used to transform the reference signal to account for the differences in gain and offset between the two signals. Lastly, the transformed references trace was subtracted from the normalized GCaMP8s trace as the final ΔF/F. The transformed reference trace was then used as UV ΔF/F. Only the experiments where the maximum percentage of DF/F exceeded 1.5 and the GCaMP8s and the UV reference correlation was below 0.6 were included for further analysis. Z-score of ΔF/F was calculated with mean and standard deviation of the entire recording session.

### Head-fixed voluntary treadmill with fiber photometry

The head-fixed locomotion was achieved with a compact low friction manually driven treadmill, originally designed by Janelia Research Campus of the HHMI, purchased from LABmaker (Berlin, Germany). Te on belt movement was tracked by a rotary encoder, which sends 0–3.3V analog output of speed and direction to an ADC port at TDT RZ10x to synchronize with photometry recordings. The speed was calibrated with 10 cm/s at 2.5V and sampled at 1k Hz. The head fixture was achieved through head fixation system along with an implantable titanium head bar from Labeotech (Montreal, Canada). Mice started treadmill habituation training one week after recovering from head-bar implant surgery to get used to walking on treadmill while head was fixed. Each habituation session lasted 20min and repeated at most twice a day for 3–4 days until mice were able to spontaneously initiate at least 20 walk bouts during a 20min session. After habituation training, a 30min fiber photometry recording session was performed for each individual mouse, while they walked on the treadmill with head fixation.

Movement versus rest time bins were defined with a 0.25 cm/s threshold on the velocity trace. Isolated movement periods with duration shorter than 0.5s or movement periods with average velocity smaller than 0.5cm/s were excluded for analysis. Because mice tended to walk slower on the treadmill, time bins were considered as rest periods only if they lasted longer than 0.8s. A movement bout initiation time was defined as velocity cross the threshold at the end of the rest period, and a movement termination time was defined as the time velocity fell below the threshold followed by a rest period. Timestamp of maximum value of z-scored GCaMP activity during 1s before and 1s after locomotion onset or offset was calculate the time of maximum activity for the respective events. Activity slope during locomotion offset was computed as the coefficient of a least-square linear regression t for the z-scored GCaMP activity during the interval of 0.5s before and after locomotion offset.

### Statistics

Data were analyzed by Prism 9 software (Graphpad) and custom code written in MATLAB (MathWorks). All statistical details for each experiment can be found in the corresponding figure legends. Data were presented as means and standard errors of the mean (SEM) or median and minimum or maximum. We assessed the statistical significance using parametric t-test and two-way analysis of variance (ANOVA). Significance was considered for test statistics with a (*) p-value of < 0.05, (**) p-value of < 0.01, (***) p-value of < 0.001.

## Data Availability

The data and codes reported in this study are available from the corresponding author upon reasonable request.
